# Comparative Demography of Skates: Life-History Correlates of Productivity and Implications for Management

**DOI:** 10.1371/journal.pone.0065000

**Published:** 2013-05-31

**Authors:** Lewis A. K. Barnett, Megan V. Winton, Shaara M. Ainsley, Gregor M. Cailliet, David A. Ebert

**Affiliations:** 1 Department of Environmental Science and Policy, University of California Davis, Davis, California, United States of America; 2 Pacific Shark Research Center, Moss Landing Marine Laboratories, Moss Landing, California, United States of America; Aristotle University of Thessaloniki, Greece

## Abstract

Age-structured demographic models were constructed based on empirical estimates of longevity and maturity for five deepwater Bering Sea skates to investigate how observed differences in life history parameters affect population growth rates. Monte Carlo simulations were used to incorporate parameter uncertainty. Estimated population growth rates ranged from 1.045 to 1.129 yr^−1^ and were lower than those reported for other Alaskan skates and most chondrichthyans. Population growth rates of these and other high-latitude skates increased with relative reproductive lifespan, but displayed no significant relationship with body size or depth distribution, suggesting that assemblage shifts may be difficult to predict for data-poor taxa. Elasticity analyses indicated that juvenile and adult survival had greater per-unit effects on population growth rates than did egg-case survival or fecundity. Population growth rate was affected more by uncertainty in age at maturity than maximum age. The results of this study indicate that if skates are deemed to be a management concern, gear modifications or depth-specific effort controls may be effective.

## Introduction

Although it has long been recognized that many elasmobranchs have life histories that may hinder the sustainability of large-scale fisheries [1], variability in productivity within and among elasmobranch taxa has only begun to be rigorously quantified. Of these taxa, it could be argued that skates have received the least research attention. However, concern for the appropriate management of skates has increased in the last decade as declines of skate populations have been detected in response to both direct and indirect fishing pressure [2], [3], [4], [5]. The emerging body of literature on skates indicates that species vary widely in life-history traits [6], which further complicates the assessment and management of mixed-species fisheries and highlights the need for species-specific data.

Eleven known species of the genus *Bathyraja* (Chondrichthyes: Rajiformes: Arhychobatidae) occur on the eastern Bering Sea (hereafter EBS) continental slope [7]. Although none of these species are currently targeted by fisheries, annual skate catches averaged between 56 and 75% of the ‘other species’ category taken as bycatch in Bering Sea and Aleutian Islands commercial bottom-trawl and longline fisheries from 1992 to 2009 [8]. Skates are considered a low-value catch, but since 1998 there has been a sharp increase in the market price of large-bodied skates, with a corresponding increase in the proportion of large-bodied skate species retained for processing [9]. Individuals that are not retained are not likely to survive because estimates of discard mortality are high (∼41–45%) [10], [11]. In addition, a pilot fishery targeting two large-bodied skate species, *Beringraja binoculata* and *Raja rhina*, has recently developed in the Gulf of Alaska [9], despite concerns that the available life-history data are insufficient to properly manage the fishery [12]. Given the current state of biological knowledge about the system, there is limited evidence to predict the population- and assemblage-level effects of fishing mortality on EBS skates.

Despite the lack of targeted take, it is plausible that current rates of indirect catch in commercial longline and trawl fisheries could affect the structure of the EBS skate assemblage. Recent research indicates that Alaskan skate species vary widely in longevity and age at maturity [13], [14], [15], [16], [17], [18], indicating high potential for community-level shifts. Evidence of directed shifts in heavily-exploited rajid assemblages has been documented in several regions, with smaller and more productive skate species becoming more dominant as larger species are disproportionately removed from communities [2], [3], [19]. In the northeast Atlantic, taxonomically-aggregated skate catch trends from the last 40 years displayed an unexpected level of consistency that disguised considerable changes in species-specific abundance [3]. Similarly, changes in EBS assemblage structure may be difficult to detect; prior to recent concerted efforts to improve the species-specificity of bycatch data in Alaska’s groundfish fisheries, approximately one-third of the observed skate catch (% by weight) reported from 2004 to 2008 was identified only as “*Bathyraja* sp.” [9].

Until recently, there were few data available to estimate species-specific life-history parameters for skates in the EBS. In the past five years, researchers have produced age, growth, and maturity estimates for eight species within the assemblage [14], [15], [16], [17], [20], [21], [22], [23]. Ebert et al. [14] used these parameter estimates to calculate population growth rates for four skate species that occur primarily on the shelf and upper slope in the EBS; however, until now, no estimates of population growth have been reported for inhabitants of the deeper portions of the slope. It is reasonable to expect that these deepwater species have lower population growth rates, given that this appears to be a general pattern within higher taxonomic groups of elasmobranchs [24], [25] and that longevity seems to scale with depth among members of the Alaskan skate assemblage [22].

Here, we aim to detect among-species trends in productivity within the EBS skate assemblage. In addition, we investigate biological and ecological correlates to population growth rates to help assess the relative productivity of species currently lacking life-history data. Towards these objectives, we used Leslie matrix models to estimate population growth rates for five slope-dwelling members of the EBS skate assemblage for which age, growth, and reproductive data have recently been reported: *Bathyraja lindbergi, B. maculata, B. taranetzi* [23], *B. minispinosa* [20], and *B. trachura* [22]. To further explore taxonomic trends in productivity, we compare the population growth rates generated from these models to those of other EBS skates, skates from other regions, and less closely related elasmobranchs. These results will provide managers with some intuition of the likely direct and indirect effects of management actions by identifying which biological processes and phases of the life-cycle are most critical for skate population growth and persistence.

## Methods

### Model Structure and Simulation

A density-independent, probabilistic, age-structured matrix modeling approach was applied [26], [27]. Vital rates were estimated from assessments of age, growth, and maturity as described below [16], [20], [22], [23]. Field sampling approval was obtained from the Institutional Animal Care and Use Committee (IACUC #501) at San Jose State University. For each species, a population projection matrix (**A**) of age-specific survival probabilities (*P_x_*, the probability of surviving from age *x* to *x* +1) and fertilities (*F_x_*) was created. The finite annual rate of population growth (*λ* = *e^r^*, where *r* is the predicted instantaneous rate of population growth per capita; *sensu* [28]) was calculated as the dominant eigenvalue of each projection matrix. Reproductive value (**v**) and stable-age distribution (**w**) were calculated as the left and right eigenvectors associated with *λ*. Two measures of generation time were calculated: the mean age of parents as calculated from the total lifetime offspring production of a parental cohort (*μ_1_*) [26] and the mean age of parents as calculated from the total offspring production of a population at the stable-age distribution (*Ā*). *Ā* was calculated from matrix elements following Cortés [27]. Other population parameters calculated were net reproductive rate (*R_0_*) [26], and rate of increase per generation (*rT*) [29]. All models were female-only with an annual time step and assumed a closed population with birth-flow reproduction.

Elasticities, or proportional sensitivities [30], [31], of *λ* were calculated to evaluate the proportional contribution of different aspects of the life cycle to *λ* following Caswell [26] as
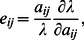
(1)where *a_ij_* is the element in row *i* and column *j* of the matrix A. Fertility, juvenile survival, and adult survival elasticities were calculated as the sum of the corresponding matrix-element elasticities. Monte Carlo simulation was used to account for uncertainty in life-history parameter estimates that may have arisen due to measurement and process error [27]. Separate independent and identically distributed (iid) probability density functions (PDFs) were created for each species to represent uncertainty in longevity (*ω*), age at maturity (*α*), age-specific survival (*S_x_*), and age-specific fecundity (*m_x_*) [27], [32], [33]. Simulations (*n* = 5,000) were performed with values of each life history parameter chosen at random from the predetermined PDFs to create replicate matrices. Separate draws for *S_x_* and *m_x_* were taken for each individual age class within a given simulation step.

To determine the effect of assuming independence in vital rates among ages, an additional set of simulations was performed with only a single draw of *S* ***_x_*** and *m* ***_x_*** per simulation step for all age classes that shared the same PDF parameters. In other words, a scenario with completely uncorrelated (or iid) vital rates among ages was compared to a scenario with very high correlation among ages. Demographic output parameters were calculated from each replicate matrix, and summarized as means and 95% confidence intervals (defined as the range of values between the 2.5^th^ and 97.5^th^ percentiles).

### Vital Rate Parameter Estimation

Mortality was expressed as age-specific annual survival rates (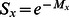, where *M_x_* is the total instantaneous rate of mortality of individuals of age *x*). Species-specific catch-at-age data were unavailable; therefore, *M_x_* was estimated with six commonly-used indirect methods based on life-history parameters (Table 1, Table S1) [34], [35], [36], [37]. Five of the methods used produce only a single mortality rate for the population, implying no difference among age classes [38], [39]. The Chen and Watanabe [40] method estimates age-specific mortality rates based on von Bertalanffy growth function parameters. Because no prior information indicated differing accuracy of specific survival estimates, a uniform PDF was selected for *S_x_*, with the range defined as the range of values obtained from all indirect estimation techniques (Table 1). Survival of the first two juvenile age classes was reduced by factors of 0.5 and 0.75, respectively, to represent additional mortality that these early-life ages are likely to incur [41], [42].

**Table 1:** Methods and indirect estimates of overall natural mortality (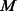) and annual survival (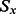, where 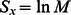) used to parameterize demographic models in this study.

Method	*Bathyraja lindbergi*		*Bathyraja maculata*		*Bathyraja minispinosa*		*Bathyraja taranetzi*		*Bathyraja trachura*	
	*M*	*S_x_*	*M*	*S_x_*	*M*	*S_x_*	*M*	*S_x_*	*M*	*S_x_*
1. 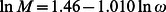 [38]	0.13	0.88	0.13	0.88	0.12	0.89	0.30	0.74	0.12	0.89
2. 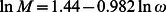 [38]	0.14	0.87	0.14	0.87	0.13	0.88	0.32	0.73	0.13	0.88
3. 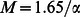 [39]	0.09	0.91	0.07	0.93	0.07	0.93	0.18	0.84	0.06	0.94
4. 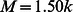 [39]	0.06	0.94	0.05	0.95	0.03	0.97	0.17	0.85	0.06	0.94
5. 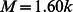 [39]	0.07	0.94	0.05	0.95	0.03	0.97	0.18	0.84	0.06	0.94
6. 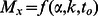 [40]	0.05–0.31	0.74–0.95	0.04–0.24	0.78–0.96	0.03–0.18	0.83–0.97	0.08–0.51	0.60–0.92	0.04–0.35	0.71–0.96

The parameters *ω* and *α* correspond to empirical estimates of longevity and the median age at maturity, respectively; *k* is the growth coefficient and *t_0_* the theoretical age when body size is zero estimated from the three-parameter von Bertalanffy growth function for each species [16], [20], [22], [23]. See Chen and Watanabe [40] for the detailed form of their age-specific mortality functions.

Embryo survival was represented by empirical estimates of egg-case predation rates in the EBS [43], [44]. Direct estimates were available for only two of the five species, *B. taranetzi* and *B. trachura*. Egg cases with similar morphologies likely experience similar levels of predation; therefore, estimates of *B. aleutica* embryo survival were used as a proxy for *B. lindbergi* and *B. maculata* [43]. Proportions of predated egg-cases across sites for five species in the EBS (*B. aleutica, B. interrupta, B. minispinosa, B. parmifera, B. trachura*) were used to bound the range of estimates for all species. Embryo survival was represented by triangular PDFs, with the most likely value for each species specified as the corresponding direct estimate or proxy (Table 2).

**Table 2:** Summary of parameter estimates and probability distribution functions (PDF) used to construct probabilistic age-structured matrix models for five deepwater Bering Sea skate species.

Species		Parameter	PDF	Minimum	Maximum	Likeliest	95% CI
*Bathyraja lindbergi*		*_ω_*	triangular	31	46	32	
		*_α_*	logistic	18	21	17.7	16.2–19.2
		*_mx_*	uniform	16	52		
		*l_e_*	triangular	0.65	0.99	0.98	
*Bathyraja maculata*		*_ω_*	triangular	31	46	32	
		*_α_*	logistic	18(21)	30(24)	22.5	21.7–23.3
		*_mx_*	uniform	16	52		
		*l_e_*	triangular	0.65	0.99	0.98	
*Bathyraja minispinosa*		*_ω_*	triangular	36	53	37	
		*_α_*	logistic	23	25	23.5	22.3–24.7
		*_mx_*	uniform	16	52		
		*l_e_*	triangular	0.65	0.98	0.87	
*Bathyraja taranetzi*		*_Ω_*	triangular	13	20	14	
		*_Α_*	logistic	8	13(10)	9.2	8.7–9.7
		*_mx_*	uniform	16	52		
		*l_e_*	triangular	0.65	0.98	0.81	
*Bathyraja trachura*		*_Ω_*	triangular	35	51	36	
		*_Α_*	logistic	22(25)	33(29)	27.5	25.8–28.1
		*_mx_*	uniform	16	52		
		*l_e_*	triangular	0.64	0.98	0.65	
							

Values in parentheses indicate minimum or maximum values used when the range generated using 95% confidence intervals was more restricted than the range bound by first and 100% maturity. *ω* = longevity (yr); *α* = median age at maturity (yr); *m_x_* = fecundity (yr^−1^); 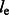 = egg case survival (yr^−1^).

A skewed triangular PDF was selected for longevity (*ω*), with the most probable value estimated to be the maximum observed age (*T*_max_). The lower range was defined as
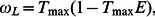
(2)where *E* is the index of average percent error (IAPE) [45] calculated using empirical data from the greatest 20% of age classes sampled by Ebert et al. [16], Maurer [23], Ainsley et al. [20], and Winton [22]. The upper range was defined as
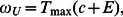
(3)Where *c* is an arbitrary constant (here *c* = 1.4) to account for the likelihood that the absolute maximum age of each species was not observed in the life history studies. Equation (3) was used because theoretical longevities calculated from von Bertalanffy growth function parameters are likely overestimates [16], [20]. The method we have chosen for establishing a maximum bound on longevity also facilitates the comparability of demographic analysis results among species [27].

Age at maturity (*α*) was simplified to a knife-edge function, with all age classes younger than the age at 50% maturity considered immature and assigned a fecundity of zero. The fecundity value for the first mature age class was halved, to reflect that only 50% of individuals would be reproducing during that year. For each species, a logistic PDF with a mean value equal to the age at 50% maturity was chosen to represent uncertainty in *α*, with lower and upper limits defined by age at first and 100% maturity [16], [20], [22], [23]. To examine the potential impact of fisheries activity on population growth rates, values of size at maturity were plotted with species-specific length-frequency data from observed commercial fishery activity in the Bering Sea and Aleutian Islands management area from 2004 to 2012 (data provided by NMFS Alaska Fisheries Science Center).

Birth rates were expressed as age-specific fecundity values (*m_x_*, where *x* = age in years). Skates are oviparous, so fecundity was defined as the number of eggs extruded female^−1^ yr^−1^. Fecundity estimates are notoriously difficult to obtain for oviparous elasmobranch species and were not available for the five species in this study; therefore, estimates from other skate species were used as a proxy. Only those fecundity estimates that were calculated with a robust method (captive parturition rate or ovarian fecundity estimates accounting for indeterminate spawning mode) were included. Mean fecundities for each skate species ranged from 32 to 104 (Table 3). Assuming a sex ratio at birth of 1∶1 [46], these values were halved to represent the number of female offspring female^−1^ year^−1^. Because no prior information indicated that the fecundity of a given skate species is a better representative of EBS skate fecundity than any other, a uniform PDF (ranging from 16 to 52) was used to represent uncertainty in fecundity for all five species.

**Table 3:** Skate fecundity data used to parameterize demographic models in this study.

Species	Mean fecundity	References
*Amblyraja radiata*	41	[80]
*Dipturus laevis*	90	[80]
*Leucoraja erinacea*	33	[81]
*Leucoraja naevus*	90	[88]
*Leucoraja ocellata*	48	[80]
*Okamejei kenojei*	86	[84]
*Raja brachyura*	65	[2], [83], [87]
*Raja clavata*	103	[82], [83], [85]
*Raja eglanteria*	60	[86]
*Raja montagui*	43	[81], [83], [87]

Many Alaskan skates have protracted and asynchronous egg-case deposition seasons [47], thus *F_x_* and *P_x_* were calculated for a birth-flow population, wherein individuals are constantly entering and leaving a given age class. To express this property in the matrix calculations, survival was approximated over the interval following Caswell [26] as
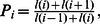
(4)where survival from *i*-1 to *i* is



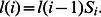
(5)Fertility coefficients were calculated following Caswell [26] as the product of averaged *m_x_* values and the survival probability for half of a time period [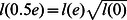
_,_ where *e* is the egg-case age class]. In mathematical terms, *F_i_* was calculated as
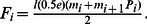
(6)


### Correlates of Population Growth Rates

Tests of correlation between population growth rates and specific life-history traits were performed to identify indicators of productivity. Estimates of population growth rates for the five species investigated here were compared with other members of the Alaskan skate assemblage as well as other high-latitude skates. Estimates for other species were obtained from the literature (Table 4). Tests were based on a *t*-statistic calculated from Pearson’s product moment correlation coefficient.

**Table 4:** Estimated finite annual population growth rates (*λ*) and possible life-history correlates to *λ* for high-latitude skates.

Species	*_λ_*	*_α_*_:*ω*_	*TL_max_* (cm)		Depth (m)	References
*Rhinoraja interrupta*	1.360	0.58	83		695	[7], [14], [46], [90]; Ebert unpubl data
*Beringraja binoculata*	1.334	0.19	244		402	[13], [14], [92], [93]
*Bathyraja aleutica*	1.252	0.53	154		809	[14], [46], [93]
*Leucoraja erinacea*	1.234	0.50	57		192	[48], [89], [94]
*Dipturus laevis*	1.221	0.24	134		800	[48], [94], [95], [96], [97]
*Raja rhina*	1.202	0.35	204		320	[7], [13], [14], [93]; Ebert unpubl data
*Amblyraja radiata*	1.197	0.58	101		859	[5], [95], [98], [99], [100]
*Leucoraja ocellata*	1.139	0.43	111		186	[48], [89], [94]
*Bathyraja taranetzi*	1.116	0.66	77		500	[7]; this study
*Bathyraja lindbergi*	1.110	0.55	97		540	[7], [93]; this study
*Bathyraja minispinosa*	1.096	0.64	90		635	[93]; this study
*Bathyraja maculata*	1.079	0.70	120		564	[7], [93]; this study
*Bathyraja trachura*	1.048	0.75	94		1169	[101], [102]; this study
*Raja clavata*	0.930	0.71	104		289	[2], [91], [103], [104], [105]

## Results

Indirect estimates of *M* ranged from 0.03 to 0.51 yr^−1^ (Table 1). Jensen’s [39] methods generally produced the lowest estimates. The age-specific Chen and Watanabe [40] method generated higher *M* estimates for younger age classes than produced by age-independent methods. Corresponding *S*_x_ values ranged between 0.77–0.95 yr^−1^ for *B. lindbergi*, 0.78–0.96 yr^−1^ for *B. maculata*, 0.83–0.97 yr^−1^ for *B. minispinosa*, 0.60–0.91 yr^−1^ for *B. taranetzi*, and 0.71–0.96 yr^−1^ for *B. trachura* (Table 1).

The projected finite rates of population increase (*λ*) were similar among the five species (Table 5). Mean *λ* ranged from 1.05 yr^−1^ (*B. trachura*) to 1.12 yr^−1^ (*B. taranetzi*), with lower confidence limits greater than 1.02 yr^−1 ^for all species. Corresponding estimates of *Ā* were longest for *B. trachura* and shortest for *B. taranetzi*; mean *μ_1_* values exhibited the same pattern. Means of *rT* and *R_o_* were least for *B. taranetzi* and *B. trachura* but similar among other species.

**Table 5:** Predicted means and 95% confidence intervals (range bounded by 2.5^th^ and 97.5^th^ percentiles) of finite annual rates of population growth (*λ*), net reproductive rates (*R_0_*), rates of increase per generation (*rT*), mean ages of females generating offspring produced by a cohort during its lifetime (*μ_1_*), and mean ages of females generating offspring produced by a population at the stable-age distribution (*Ā*).

Species		*λ*	*R0*	*rT*	*μ1*	*Ā*
	2.5%	1.090	8.51	2.04	23.71	21.54
*Bathyraja lindbergi*	**mean**	**1.112**	**13.18**	**2.47**	**25.30**	**22.61**
	97.5%	1.132	18.95	2.91	27.35	24.35
	2.5%	1.063	5.45	1.57	26.64	24.71
*Bathyraja maculata*	**mean**	**1.081**	**9.12**	**2.05**	**28.53**	**26.33**
	97.5%	1.100	13.99	2.54	30.79	28.08
	2.5%	1.080	10.46	2.25	29.27	27.32
*Bathyraja minispinosa*	**mean**	**1.097**	**16.82**	**2.73**	**31.33**	**28.80**
	97.5%	1.114	25.10	3.22	33.93	30.60
	2.5%	1.063	2.13	0.60	11.30	9.31
*Bathyraja taranetzi*	**mean**	**1.119**	**3.98**	**1.13**	**12.25**	**10.36**
	97.5%	1.170	6.33	1.60	13.47	11.52
	2.5%	1.027	2.38	0.87	30.08	29.44
*Bathyraja trachura*	**mean**	**1.048**	**4.86**	**1.53**	**32.54**	**31.81**
	97.5%	1.068	8.26	2.11	35.47	34.43

Values were estimated from 5,000 Monte Carlo simulations.

**Figure 1:**
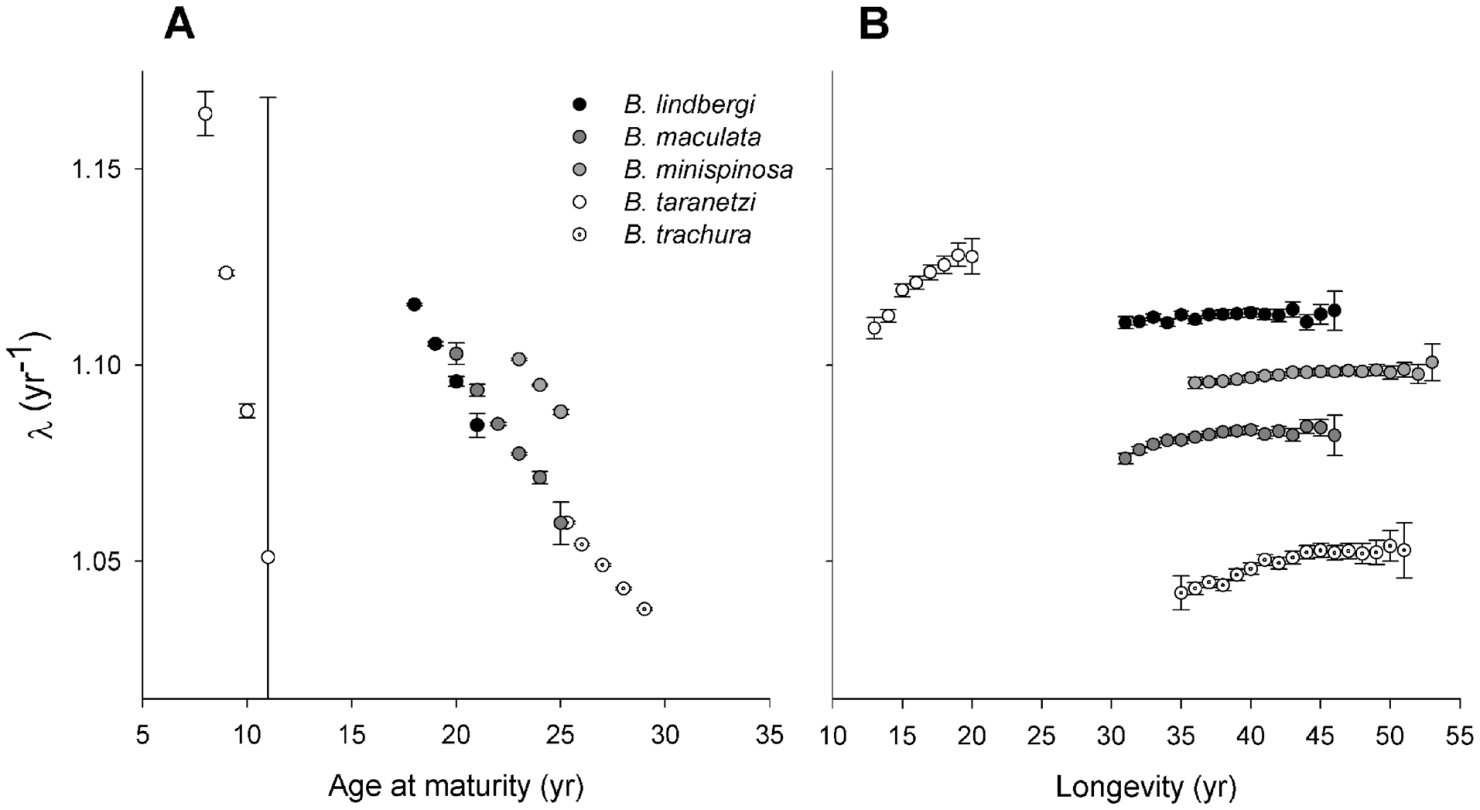
Influence of age at maturity and longevity on finite population growth rates (*λ*). Mean estimates were produced using probabilistic, age-structured matrix models based on empirical estimates of longevity and maturity for five Bering Sea skate species: *Bathyraja lindbergi, B. maculata, B. minispinosa, B. taranetzi,* and *B. trachura*. Variability in the estimated age at maturity (A) affected *λ* with greater per-unit magnitude than variation in longevity (B). Error bars indicate 95% confidence intervals.

**Figure 2:**
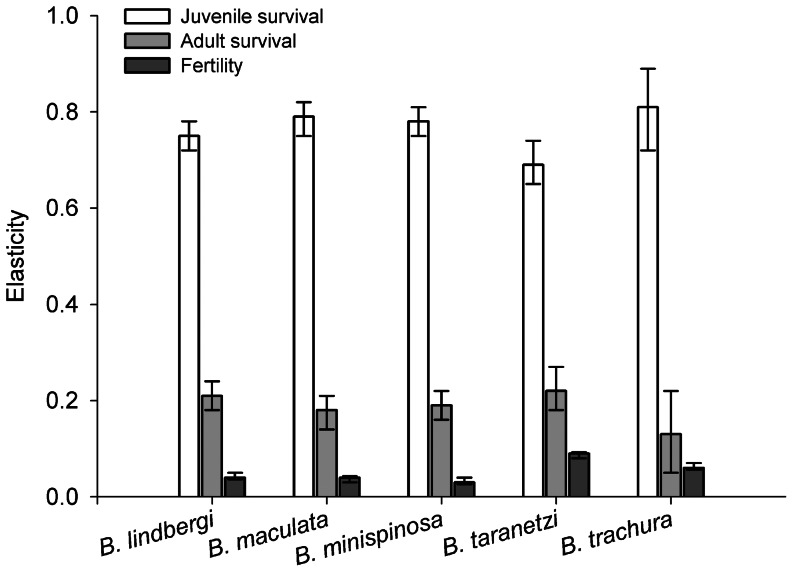
Elasticity analysis. Predicted means and 95% confidence intervals (range bounded by 2.5^th^ and 97.5^th^ percentiles) of elasticities for five Bering Sea skate species. Mean values were estimated from 5,000 Monte Carlo simulations assuming independent and identically distributed vital rates among age classes.

Population growth rates were more sensitive to variation in age at maturity than any other parameter, with only minor changes in response to variation in longevity (Figure 1). Elasticity patterns were similar among all five species. Juvenile survival elasticities were the greatest for all species and ranged from a mean of 0.69 for *B. taranetzi* to 0.81 for *B. trachura* (Figure 2). Mean fertility elasticities were the least, ranging from 0.03 for *B. minispinosa* to 0.09 for *B. taranetzi.* Expressing these elasticities as ratios indicates that in order to compensate for as little as a 10% decrease in juvenile survival, an 81% (*B. taranetzi*) to 226% (*B. minispinosa*) increase in fertility or egg case survival would be required to return populations to their initial growth rates, depending on the species in question. Similarly, a 10% decrease in adult survival would require increases in fertility or egg case survival ranging from 21% (*B. trachura*) to 55% (*B. minispinosa*). Elasticity ratios of adult to juvenile survival were smaller; a 10% decrease in adult survival could be compensated for by increases in juvenile survival ranging from approximately 2% to 3% for all species.

**Figure 3:**
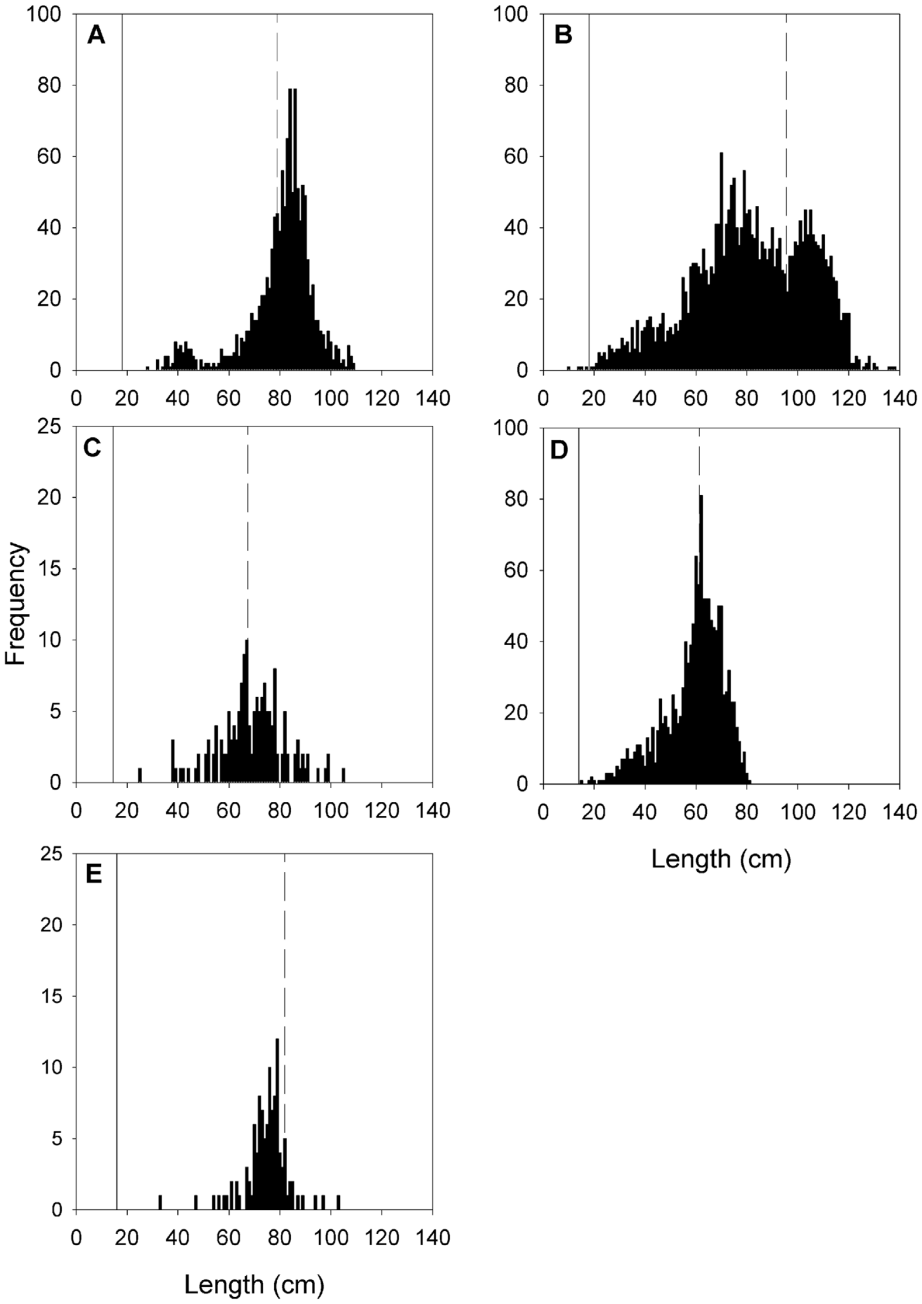
Length frequency distributions of skate bycatch in the eastern Bering Sea. Data were obtained from observers (National Marine Fisheries Service, Alaska Fisheries Science Center) during commercial fishery activity in the Bering Sea and Aleutian Islands management area from 2004 to 2012. Estimated size at birth (solid line) and size at maturity (dashed line) are indicated for each species: *Bathyraja lindbergi* (A), *B. maculata* (B), *B. minispinosa* (C), *B. taranetzi* (D), and *B. trachura* (E).

Examination of length-frequency data from commercial fisheries in the EBS indicated that all five species are captured as both juveniles and adults (Figure 3). The smallest *B. lindbergi, B. trachura* and *B. minispinosa* observed in the catch were only slightly larger than the estimated size at hatching. In general, the dominant mode present in the catch was near the size at maturity. One exception to this pattern was found in the catch of *B. maculata*, which exhibited two modes at sizes slightly smaller and larger than the size at maturity, which were separated by a local minimum at the size at maturity itself.

### Effects of Correlated Vital Rate Elements

Simulations with correlation in vital rates among ages in a given year produced similar mean outcomes as the base case (Table 6), but substantially increased the variability of these estimates (95% confidence limits for *λ* were as low as 1.01 yr^−1^ for *B. trachura* and as high as 1.22 yr^−1^ for *B. taranetzi*; Table 6). Compared to the fully uncorrelated scenario, mean *λ* and *rT* were marginally decreased, but variability was substantially increased. Both mean and variance of *R_0_* were substantially increased. Neither means nor variances of *Ā*, *μ_1_* or any of the elasticities (Figure S1) were appreciably changed.

**Table 6:** Predicted means and 95% confidence intervals (range bounded by 2.5^th^ and 97.5^th^ percentiles) of finite annual rates of population growth (*λ*), net reproductive rates (*R_0_*), rates of increase per generation (*rT*), mean ages of females generating offspring produced by a cohort during its lifetime (*μ_1_*), and mean ages of females generating offspring produced by a population at the stable-age distribution (*Ā*).

Species		*λ*	*_R0_*	*_rT_*	*_μ1_*	*_Ā_*
	2.5%	1.056	3.70	1.22	23.68	21.30
*Bathyraja lindbergi*	**mean**	**1.110**	**15.01**	**2.44**	**25.30**	**22.62**
	97.5%	1.164	38.73	3.67	27.66	24.41
	2.5%	1.022	1.82	0.55	26.65	24.25
*Bathyraja maculata*	**mean**	**1.079**	**11.51**	**2.01**	**28.52**	**26.32**
	97.5%	1.137	36.76	3.53	31.03	28.53
	2.5%	1.039	3.15	1.08	29.13	26.82
*Bathyraja minispinosa*	**mean**	**1.096**	**22.47**	**2.70**	**31.37**	**28.81**
	97.5%	1.151	75.00	4.42	34.69	31.03
	2.5%	1.013	1.17	0.12	11.36	9.24
*Bathyraja taranetzi*	**mean**	**1.116**	**4.27**	**1.10**	**12.24**	**10.34**
	97.5%	1.218	10.41	2.03	13.43	11.57
	2.5%	1.008	1.31	0.27	30.07	29.42
*Bathyraja trachura*	**mean**	**1.046**	**5.11**	**1.46**	**32.52**	**31.81**
	97.5%	1.083	13.10	2.57	35.46	34.49

Values were estimated from 5,000 Monte Carlo simulations with perfect correlation in vital rates among age classes with the same probability distributions.

### Correlates of Population Growth Rates

**Figure 4:**
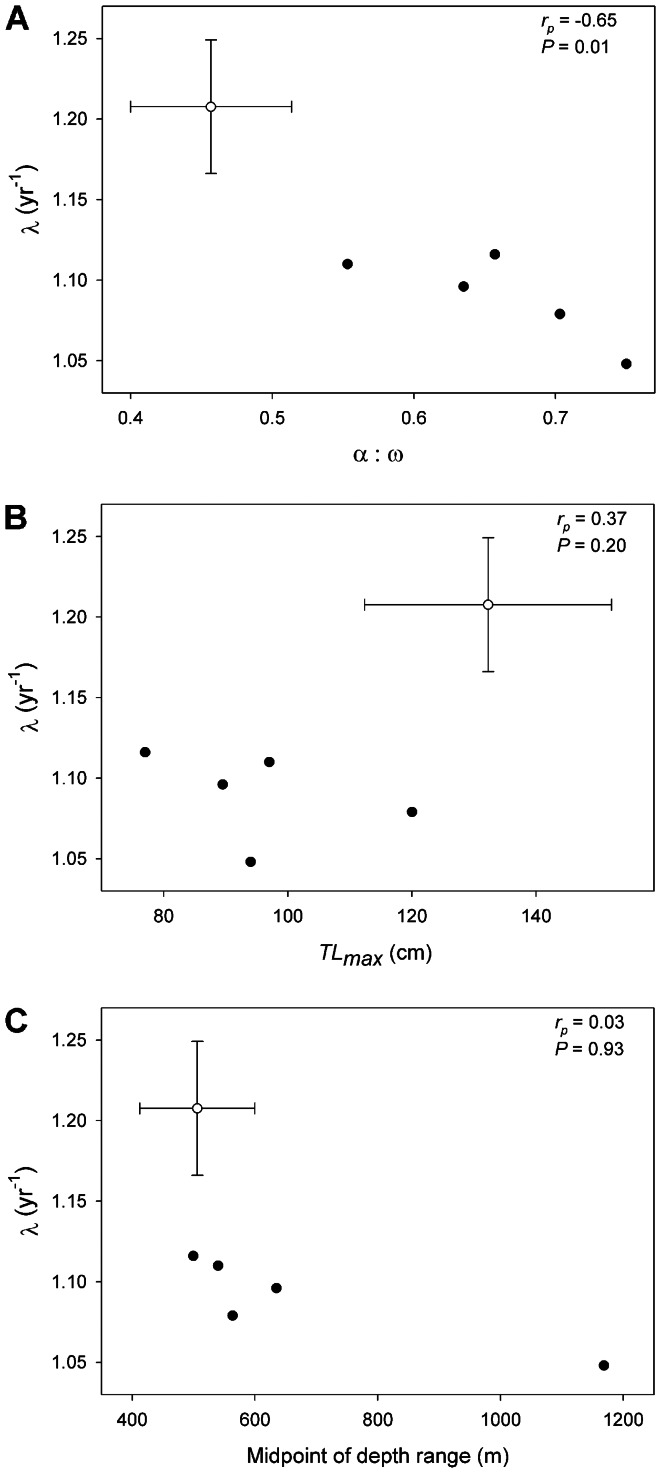
Correlates of population growth rates in high latitude skates. Mean finite rates of population growth (*λ*) predicted from probabilistic, age-structured matrix models in relation to the proportion of lifespan devoted to maturation [the ratio of age at maturity (α) to longevity (ω)] (A), maximum total length (*TL_max_*; B), and midpoint of depth range (C). Note that higher values of α:ω, reported as a proportion, reflect a shorter reproductive lifespan relative to the total lifespan. Data are shown for each of the five species analyzed in this study, in comparison to the mean and variance from nine other high-latitude skates (Table 5). Pearson’s product-moment correlation coefficients and related *P*-values are shown for each relationship. Error bars indicate 95% confidence intervals (among-species).

**Figure 5:**
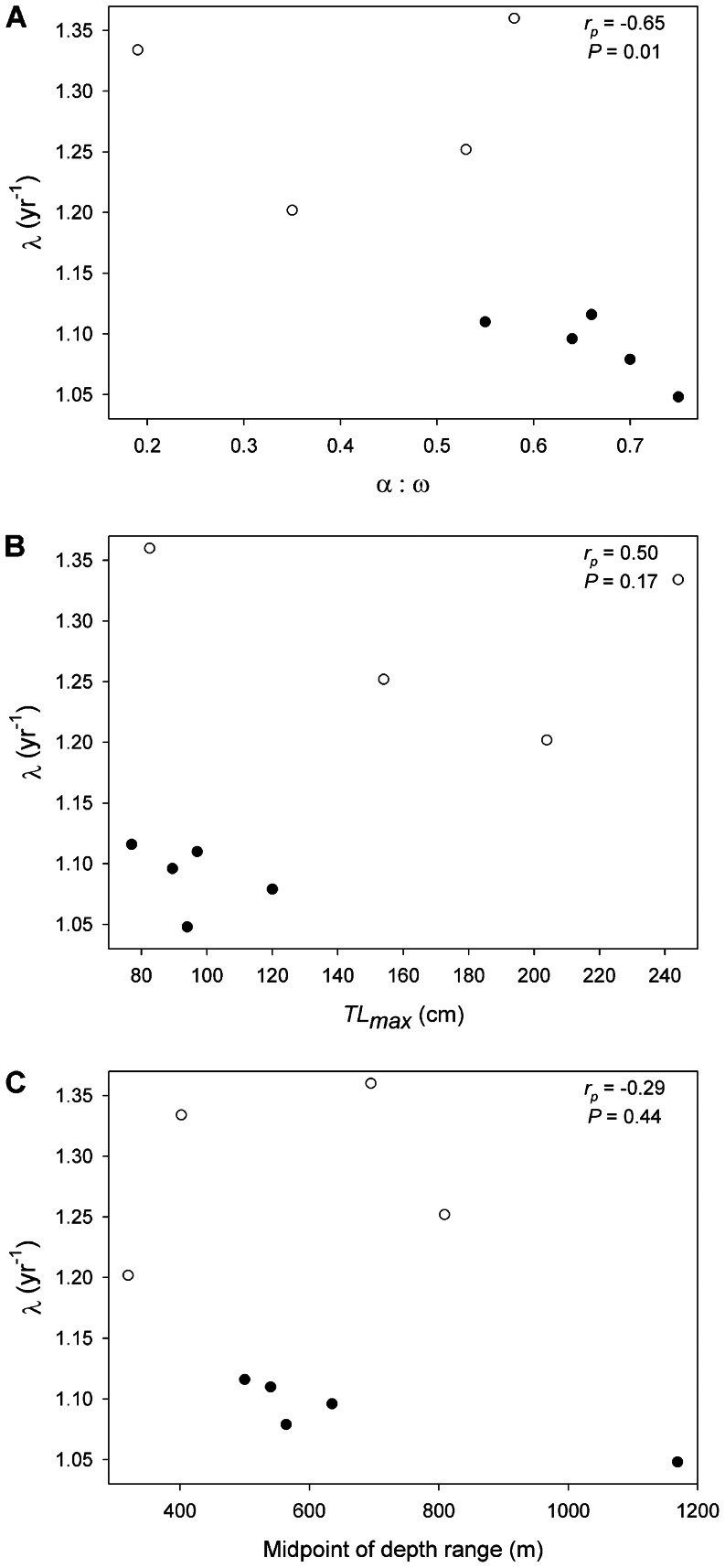
Correlates of population growth rates in Alaskan skates. Mean finite rates of population growth (*λ*) predicted from probabilistic, age-structured matrix models in relation to the proportion of lifespan devoted to maturation [the ratio of age at maturity (α) to longevity (ω)] (A), maximum total length (*TL_max_*; B), and midpoint of depth range (C). Note that higher values of α:ω, reported as a proportion, reflect a shorter reproductive lifespan relative to the total lifespan. Data are shown for each of the five species analyzed in this study (filled circles), in comparison to the four other species of Alaskan skates for which data were available (open circles; Table 5). Pearson’s product-moment correlation coefficients and related *p*-values are shown for each relationship.

Among high-latitude skates for which data were available (Table 4), population growth rates were negatively correlated with the ratio of age at maturity to longevity (Figure 4; Pearson’s product-moment correlation, *r_p_* = -0.65, *n* = 14, *P* = 0.01). In contrast, there was no significant correlation between population growth rate and maximum total length (*r_p_* = 0.37, *n* = 14, *P* = 0.19) or the midpoint of the depth range (*r_p_* = 0.03, *n* = 14, *P = *0.93). Similarly, within the EBS skate assemblage population growth rates were negatively correlated with the ratio of age at maturity to longevity (Figure 5; *r_p_* = -0.69, *n* = 9, *P* = 0.04) and were not significantly correlated with maximum total length (*r_p_* = 0.50, *n* = 9, *P* = 0.17) or the midpoint of the depth range (*r_p_* = -0.29, *n* = 9, *P = *0.44). Note that the ratio of age at maturity to longevity increases as the number of reproductive years becomes lower relative to the total lifespan; thus this metric is an inverse proxy for reproductive lifespan (i.e., higher values generally indicate a shorter window of lifetime reproduction).

Among high-latitude skates for which data were available (Table 4), population growth rates were negatively correlated with the proportion of the lifespan devoted to maturation, or the ratio of age at maturity to longevity (Figure 4; Pearson’s product-moment correlation, *r_p_* = -0.65, *n* = 14, *P* = 0.01). In contrast, there was no significant correlation between population growth rate and maximum total length (*r_p_* = 0.37, *n* = 14, *P* = 0.19) or the midpoint of the depth range (*r_p_* = 0.03, *n* = 14, *P = *0.93). Similarly, within the EBS skate assemblage population growth rate was negatively correlated with the proportion of lifespan devoted to maturation (Figure 5; *r_p_* = -0.69, *n* = 9, *P* = 0.04) and was not significantly correlated with maximum total length (*r_p_* = 0.50, *n* = 9, *P* = 0.17) or the midpoint of the depth range (*r_p_* = -0.29, *n* = 9, *P = *0.44). Note that the proportion of the lifespan devoted to maturation is equivalent to 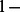 [relative reproductive lifespan], where the relative reproductive lifespan is reproductive lifespan scaled to longevity. Thus, the above results indicate that population growth rates increased with the relative reproductive lifespan.

## Discussion

Elasmobranch life histories have been demonstrated to fall along a “fast-slow” continuum, with “slow” species defined as those with later age at maturity, longer generation times, and lower population growth rates than “fast” species [4], [27]. The relative position of skates along this life-history continuum remains unclear because few demographic analyses have been performed on this group. Of the skate species analyzed herein, *B. taranetzi* was the most productive; this species had the highest predicted population growth rate and shorter generation time than the other species. *Bathyraja lindbergi* and *B. maculata* were the next most productive, with the former perhaps slightly more robust to perturbations in mortality because of a shorter generation time than that of *B. maculata*. *Bathyraja trachura* is perhaps the least productive of the species analyzed here, primarily because it has the longest generation time.

Among studies that have incorporated uncertainty in life-history parameters for other elasmobranchs, *λ* values estimated for the five species herein were substantially lower than the midpoint of the range (*λ* = 1.25 yr^−1^), but similar to the median (*λ* = 1.08 yr^−1^) [14], [27], [33], [48], [49], [50], [51], [52], [53], [54]. These results are contrary to the results of prior cross-taxonomic comparisons, which placed the Rajiformes as the chondrichthyan order least vulnerable to extinction [24] and the most likely to recover from overexploitation [25]. Using the Musick [55] criteria based on productivity–as measured by population growth rate–all five skates examined in this study are classified as vulnerable to population depletion by fishing mortality.

In combination with estimated population growth rates, evaluation of other demographic parameter estimates also indicates that the species analyzed here have moderately “slow” life histories compared to other elasmobranchs and are substantially “slower” than other Alaskan skates. Mean generation times for all species except *B. taranetzi* were higher than other Alaskan skates [14]. Compared to other elasmobranchs, values of generation time for *B. taranetzi* were close to the median (*Ā* = 11.20 yr), but less than the midpoint (*Ā* = 29.25 yr) of the range [14], [27], [33], [49], [50], [52]; for the other four species, values of *Ā* were in the upper end of the range. Mean rates of increase per generation (*rT*) were lower than other Alaskan skates [14] but higher than those of many other elasmobranchs [33], [56], with the exception of *B. taranetzi*. Values of *R_0_* were lower than those of most other Alaskan skates [14], with some similarity between *B. aleutica* (mean *R_0_* = 23.26; [14]) and *B. minispinosa* (mean *R_0_* = 16.82; Table 5). In summary, skate life histories likely exhibit a similar continuum to that of sharks, with substantial interspecific variation present even within the North Pacific basin. These results further emphasize the idea that this type of fast-slow continuum occurs within many lower taxonomic groups [57], and that it is often not appropriate to generalize a specific life-history strategy to an entire family.

Variability in *α* often affects *λ* with greater per-unit magnitude than other vital rates in elasmobranchs [4], [27]; there is often enough uncertainty in this parameter to cause great uncertainty in estimates of *λ*, e.g. [58]. Population growth rates for the five species investigated herein were highly sensitive to this parameter estimate, as exemplified by the difference in the absolute change in *λ* across the range of parameter uncertainty in *α* relative to *ω* (Figure 1). Age at maturity was particularly influential in the case of the shorter-lived *B. taranetzi*. Similarly, the effect of differing *ω* on *λ* was greater in *B. taranetzi* than the other four species. These interspecific differences were likely caused by the presence of a higher relative proportion of individuals remaining in the reproductive and ultimate age classes in *B. taranetzi*.

Elasticity analyses indicated that population growth rates were more influenced by juvenile and adult survival than the combination of egg case survival and fecundity (encompassed within the fertility elasticity; Figure 2), supporting this as a general pattern for many long-lived vertebrates [4], [27], [59], [60]. Therefore, all five species follow the expected pattern proposed by Cortés [27], who found that juvenile survival elasticities were the highest for all shark species with *α* ≥5 yr. Predicted rank order of elasticity values was similar to long-lived elasmobranchs [27] and sea turtles [59]. Juvenile survival elasticities estimated in this study represent some of the highest values estimated for any vertebrate, whereas fertility elasticities were on the extreme low end of this spectrum [59], [61]. Concordantly, adult survival elasticities were quite low relative to other vertebrates. Juvenile survival and fertility elasticities were not as extreme for *B. taranetzi* as for the other four species. Adult survival elasticities were most extreme for *B. trachura*; values for the other four species were moderately low compared to all elasmobranchs [median = 0.31; range = (0.12–0.51)] [14], [27], [33], [49], [50], [51], [52], [53].

Given the limited potential of elasmobranchs to increase adult survival or fecundity, exploitation compensation would most likely be exhibited through increases in egg case or juvenile survival, or through decreases in age at maturity over time [4], [27]. The results reported herein indicate that density-dependent compensation in the form of increased egg case survival would not substantially increase *λ*, particularly for *B. lindbergi*, *B. maculata*, and *B. minispinosa*. Additionally, there is evidence that embryonic survival during the egg case stage is positively correlated with density in skates [44], making increased egg case survival an even less likely compensation mechanism. In contrast, ratios of adult to juvenile survival indicate that compensation is more likely to occur through increased juvenile survival due to reduced intraspecific competition at lower population densities.

### Data Quality and the Status of the Best Available Science in Elasmobranch Demography

It is important to note that the demographic parameter estimates reported herein are a first approximation and will be improved as life-history data continue to be collected. The reliability of the results of demographic models is determined by the quality of the vital rate estimates incorporated; the more robust and precise the parameter estimates and the assumptions they rely upon, the more reliable any potential management strategy based upon the results. For example, because sample sizes used for age estimation of each species were likely not large enough to include the absolute oldest individuals in the population, maximum observed ages may underestimate the species’ true longevity. However, maximum ages are likely not far off those empirically estimated; therefore, the application of a correction factor was used to produce longevity estimates within reasonable bounds. Additionally, age estimates were based on the assumption of annual band pair formation and have not been directly validated in these species [16], [20], [22], [23]. Considering the importance of accurate age estimates for management, direct validation of age estimates is imperative. Although the models presented were developed using the best available information, the uncertainty in longevity and the lack of species-specific fecundity data dictates that these results should not be used for applied purposes without appropriate precaution (see Text S1 for additional caveats and empirical needs).

Although there have been recent calls to incorporate density-dependent effects into demographic models [28], [62], the data necessary to parameterize these effects is sorely lacking. In addition, some of the techniques proposed assume that there is no fishing mortality on juveniles [63], which is violated in the case of EBS skates [8], as indicated by the length-frequency distribution of skates taken as bycatch in commercial fisheries in the EBS (Fig. 3). Compensation is expected to occur primarily through increased juvenile survival at low population sizes [64], yet creating a realistic representation of this effect is rarely possible given the current quality of chondrichthyan data. Models that have attempted to incorporate density-dependent effects have demonstrated a limited magnitude of potential compensatory effects [65]. Proper representation would require collection of repeated estimates of juvenile survival over time or space, coupled with concurrent density estimates.

### Life-history Correlates of Population Growth Rate

Results of both comparisons among all high-latitude skates (Fig. 4) and comparisons within the EBS region (Figure 5) support neither the paradigm of a negative correlation of population growth rate with body size [66], nor the paradigm of correlation with depth [24], [25], [67]. These results indicate that the nature of potential shifts in community structure of skates will not be easily predicted by body size, as has been argued for the North Atlantic skate assemblage [66]. For the eastern Scotian Shelf skate assemblage, McPhie and Campana [5] found that the species with the highest ratios of age at maturity to maximum age had the lowest predicted population growth rates, and that this was a better predictor of population growth rate than was body size. Including the species analyzed here, data for high-latitude skates supports the ratio between age at maturity and longevity as the best indicator of population growth rate (Figs. 4, 5). This does not necessarily invalidate the use of body size as an indicator of productivity or vulnerability when age data are not available, as these sets of metrics often covary. Body size may indeed explain different forms of variation in life histories than those encompassed by age metrics [68].

The greater importance of these age metrics relative to body size has also been identified at higher taxonomic scales among chondrichthyans [69], teleosts and mammals [70]. This result is intuitive; the earlier in life that a species begins reproducing and the more years it reproduces increases its expected lifetime reproductive output. This could be offset by interspecific differences in fecundity or patterns of natural mortality, but the analyses here and elsewhere suggest that this is not the case. Finally, it must be cautioned that although the direction of these relationships are likely robust, the specific form of these relationships should not be taken as gospel given the relatively low sample sizes in these correlation tests.

Across much broader taxonomic scales than those investigated here, theory and data have shown that there are invariant relationships between age at maturity and longevity (or reproductive lifespan) within related taxa [71]. Thus, this metric has been previously established as useful for characterizing life-history patterns [72], [73]; it reflects the tradeoff between the mortality risk of delaying maturity and the reproductive benefits of increased fecundity with larger body size [74]. Our results add support to the importance of this quantity as a metric for synthesizing how suites of life history traits combine to determine population growth rate.

### Management Implications

Since 2011, skates in the EBS have been managed as a separate aggregate complex rather than as a conglomeration with dissimilar taxa [8]. Though this is certainly an improvement over previous practices, skate assemblages in other regions have experienced alterations in abundance, size and age structure, and species composition as a result of both direct and indirect fishing effort over relatively short timescales [2], [3], [19]. To date, demographic analyses have been conducted on nine of the fifteen members of the Alaskan skate assemblage (this study; [14]). Resulting estimates of demographic parameters indicate that the five species investigated herein are among the least productive species in the assemblage and may experience more rapid declines than other species as the result of exploitation (Table 4). Among the five species analyzed here, it appears that commercial fisheries most often encounter *B. maculata*, with *B. lindbergi* and *B. minispinosa* a close second; *B. taranetzi* and *B. trachura* are caught in much lower numbers (Fig. 3). The combination of evidence indicates that *B. maculata* is the most likely to decline in abundance, given the relatively low predicted rate of population growth and high fisheries-encounter and retention rates [9]. Although considerable measures have been taken in recent years to record species-specific bycatch data in Alaska’s groundfish fisheries, current predictions of potential assemblage-wide shifts would be speculative, given the sparseness of this dataset and limited knowledge of the differences in the size- and depth-selectivity of the region’s fisheries.

The results of the present study provide valuable information to inform decisions regarding management of the skate assemblage in the EBS. Length-frequency data collected by observers in the EBS and Aleutian Islands indicates that both juveniles and adults of all five species are taken as bycatch in the regions’ fisheries and that skates are susceptible to commercial gear at extremely young ages (Figure 3). Given the relative similarity of hatching size among the five species of interest, the presence of newly-hatched *B. maculata* and *B. taranetzi* in the catch indicates that the entire assemblage is likely susceptible to gear from the time of hatching. There is also evidence that egg-cases are occasionally caught in large quantities in Bering Sea groundfishery bottom trawls (North Pacific Fisheries Groundfish Observer Program unpubl data, as cited in [47]); therefore, these five species are likely exploited at all stages of their lifecycle. In addition to the potentially negative effects of early age at first recruitment on sustainable catch rates [65], elasticity analyses indicate that population growth rates of these five species are more affected on a per-unit basis by changes in juvenile survival rather than adult or egg case survival. Thus, efforts directed at regulating juvenile mortality would likely be the most effective approach if mitigation of population depletion becomes a management concern.

Although elasticities do not provide a sufficient basis for management on their own [27], [59], they do facilitate prioritization of management efforts. Trawl survey data indicate that several areas along the EBS shelf serve as nursery sites for bathyrajid species [47]. Although elasticity analyses indicate that changes in embryonic and adult survival do not have as large of a per-unit influence on population growth rates as does juvenile survival, protection of nursery sites where egg cases and adults are aggregated [47] is nevertheless likely to be important for population persistence, particularly when considering the sheer magnitude of potential effects of trawl gear on such sites. As such, it would benefit fisheries managers to consider how management actions directed at one life stage affect vital rates at other life stages [27].

If managers determine that fishery impacts are imposing unacceptably negative effects on skate populations in the region, depth closures could be effective. Rajids are often distributed bathymetrically according to size, e.g. [75]; thus, permanent or temporary depth/area closures could be implemented to reduce juvenile mortality and stabilize age at maturity schedules. For skates, it is often assumed that all ages are potentially vulnerable to fishery mortality [8], with a slight increase in selectivity with size [76]. The bycatch data presented here indicate that selectivity for most EBS skates (with the exception of *B. maculata*) reaches a maximum near the size at maturity and perhaps declines at larger sizes (Figure 3). This pattern is troubling because it is indicative of directional selection on size and age at maturity. The results of the elasticity analysis presented here suggest this form of selection on maturation may lead to dramatic changes in population growth rates.

Within the EBS skate assemblage, body size is negatively correlated with depth ([47]; Ebert unpubl data). Therefore, restricting fishing at mid-depths and concentrating fishing at shallower and deeper depths could create a situation that approximates a *de facto* slot fishery, thereby reducing artificial selection for change in age at maturity. Currently, the skates with the deepest distributions off of Alaska have quite low bycatch rates [9], but if the fleets shift their effort and begin fishing more intensively in the deeper depth strata, these species would benefit greatly from depth restrictions, particularly in areas of high egg-case densities [47]. Based on the available length-frequency data of skate bycatch in the region, juvenile mortality from trawl gear could be substantial if fishing occurs in or around nursery sites. With innovation it is possible that bycatch reduction devices could be used to accomplish the same objectives of size-selective harvest [77], [78]. These fisheries management recommendations are more or less in line with previous suggestions for chondrichthyans [60], [64], including those provided through density-dependent analyses [65], [79].

## Supporting Information

Figure S1 **Elasticity analysis for simulations incorporating correlation in vital rates.** Predicted means and 95% confidence intervals (range bounded by 2.5^th^ and 97.5^th^ percentiles) of elasticities for five Bering Sea skate species. Mean values were estimated from 5,000 Monte Carlo simulations assuming perfect correlation in vital rates among age classes with the same probability distributions.
(DOCX)

Table S1 **Additional natural mortality estimates.** Age-specific estimates of natural mortality (*M_x_*) and annual survival (*S_x_*, where *S_x_ = *ln *M_x_*) used to parameterize demographic models in this study. Calculations follow Chen and Watanabe [41].
(DOCX)

Text S1 **Additional caveats and empirical needs.** Additional text pertaining to the discussion section *Data quality and the status of the best available science in elasmobranch demography*.
(DOCX)
