# Supplementary material for: Comparative Demography of Skates: Life-History Correlates of Productivity and Implications for Management
Source: PLoS One. 2013 May 31;8(5):e65000. doi: 10.1371/journal.pone.0065000 (PMC3669027; doi:10.1371/journal.pone.0065000)
Supplement: Figure S1 — Elasticity analysis for simulations incorporating correlation in vital rates. Predicted means and 95% confidence intervals (range bounded by 2.5th and 97.5th percentiles) of elasticities for five Bering Sea skate species. Mean values were estimated from 5,000 Monte Carlo simulations assuming perfect correlation in vital rates among age classes with the same probability distributions. (DOCX) [file pone.0065000.s001.docx]

**Figure S1.** **Elasticity analysis for simulations incorporating correlation in vital rates.** Predicted means and 95% confidence intervals (range bounded by upper 2.5th and lower 97.5th percentiles) of elasticities. Values were estimated from 5,000 realizations of a simulation with perfect correlation among age classes in vital rates with the same probability distributions.
