# Supplementary material for: Comparative Demography of Skates: Life-History Correlates of Productivity and Implications for Management
Source: PLoS One. 2013 May 31;8(5):e65000. doi: 10.1371/journal.pone.0065000 (PMC3669027; doi:10.1371/journal.pone.0065000)
Supplement: Table S1 — Additional natural mortality estimates. Age-specific estimates of natural mortality (Mx) and annual survival (Sx, where Sx = ln Mx) used to parameterize demographic models in this study. Calculations follow Chen and Watanabe [41]. (DOCX) [file pone.0065000.s002.docx]

**Table S1. Additional natural mortality estimates.** Age-specific estimates of natural mortality (*M*_x_) and annual survival (*S*_x_, where ) used to parameterize demographic models in this study. Calculations follow Chen and Watanabe [2]

| *Age* | *Bathyraja lindbergi* | | *Bathyraja maculata* | | *Bathyraja minispinosa* | | *Bathyraja taranetzi* | | *Bathyraja trachura* | |
| --- | --- | --- | --- | --- | --- | --- | --- | --- | --- | --- |
|  | *M*_x_ (yr^-1^) | *S*_x_ (yr^-1^) | *M*_x_ (yr^-1^) | *S*_x_ (yr^-1^) | *M*_x_ (yr^-1^) | *S*_x_ (yr^-1^) | *M*_x_ (yr^-1^) | *S*_x_ (yr^-1^) | *M*_x_ (yr^-1^) | *S*_x_ (yr^-1^) |
| 0 | 0.26 | 0.77 | 0.24 | 0.78 | 0.18 | 0.83 | 0.51 | 0.60 | 0.35 | 0.71 |
| 1 | 0.21 | 0.81 | 0.20 | 0.82 | 0.16 | 0.85 | 0.37 | 0.69 | 0.27 | 0.77 |
| 2 | 0.18 | 0.83 | 0.17 | 0.84 | 0.14 | 0.87 | 0.30 | 0.74 | 0.22 | 0.80 |
| 3 | 0.16 | 0.85 | 0.15 | 0.86 | 0.12 | 0.88 | 0.25 | 0.78 | 0.19 | 0.83 |
| 4 | 0.14 | 0.87 | 0.14 | 0.87 | 0.11 | 0.89 | 0.22 | 0.80 | 0.16 | 0.85 |
| 5 | 0.13 | 0.88 | 0.12 | 0.88 | 0.10 | 0.90 | 0.20 | 0.82 | 0.14 | 0.87 |
| 6 | 0.12 | 0.89 | 0.11 | 0.89 | 0.10 | 0.91 | 0.19 | 0.83 | 0.13 | 0.88 |
| 7 | 0.11 | 0.89 | 0.11 | 0.90 | 0.09 | 0.91 | 0.17 | 0.84 | 0.12 | 0.89 |
| 8 | 0.10 | 0.90 | 0.10 | 0.91 | 0.08 | 0.92 | 0.16 | 0.85 | 0.11 | 0.89 |
| 9 | 0.10 | 0.91 | 0.09 | 0.91 | 0.08 | 0.92 | 0.16 | 0.86 | 0.10 | 0.90 |
| 10 | 0.09 | 0.91 | 0.09 | 0.92 | 0.07 | 0.93 | 0.15 | 0.86 | 0.10 | 0.91 |
| 11 | 0.09 | 0.92 | 0.08 | 0.92 | 0.07 | 0.93 | 0.14 | 0.87 | 0.09 | 0.91 |
| 12 | 0.08 | 0.92 | 0.08 | 0.92 | 0.07 | 0.93 | 0.14 | 0.87 | 0.09 | 0.92 |
| 13 | 0.08 | 0.92 | 0.08 | 0.93 | 0.06 | 0.94 | 0.08 | 0.92 | 0.08 | 0.92 |
| 14 | 0.08 | 0.92 | 0.07 | 0.93 | 0.06 | 0.94 | 0.08 | 0.92 | 0.08 | 0.92 |
| 15 | 0.08 | 0.93 | 0.07 | 0.93 | 0.06 | 0.94 | 0.08 | 0.92 | 0.08 | 0.93 |
| 16 | 0.07 | 0.93 | 0.07 | 0.93 | 0.06 | 0.94 | 0.09 | 0.92 | 0.07 | 0.93 |
| 17 | 0.07 | 0.93 | 0.07 | 0.94 | 0.06 | 0.95 | 0.10 | 0.91 | 0.07 | 0.93 |
| 18 | 0.07 | 0.93 | 0.06 | 0.94 | 0.05 | 0.95 | 0.11 | 0.89 | 0.07 | 0.93 |
| 19 | 0.07 | 0.94 | 0.06 | 0.94 | 0.05 | 0.95 | 0.15 | 0.86 | 0.07 | 0.94 |
| 20 | 0.07 | 0.94 | 0.06 | 0.94 | 0.05 | 0.95 | 0.27 | 0.76 | 0.07 | 0.94 |
| 21 | 0.06 | 0.94 | 0.06 | 0.94 | 0.05 | 0.95 |  |  | 0.06 | 0.94 |
| 22 | 0.06 | 0.94 | 0.06 | 0.94 | 0.05 | 0.95 |  |  | 0.06 | 0.94 |
| 23 | 0.06 | 0.94 | 0.06 | 0.95 | 0.05 | 0.95 |  |  | 0.06 | 0.94 |
| 24 | 0.06 | 0.94 | 0.06 | 0.95 | 0.04 | 0.96 |  |  | 0.06 | 0.94 |
| 25 | 0.06 | 0.94 | 0.05 | 0.95 | 0.04 | 0.96 |  |  | 0.06 | 0.94 |
| 26 | 0.06 | 0.94 | 0.05 | 0.95 | 0.04 | 0.96 |  |  | 0.06 | 0.94 |
| 27 | 0.06 | 0.94 | 0.05 | 0.95 | 0.04 | 0.96 |  |  | 0.06 | 0.95 |
| 28 | 0.06 | 0.95 | 0.05 | 0.95 | 0.04 | 0.96 |  |  | 0.05 | 0.95 |
| 29 | 0.06 | 0.95 | 0.05 | 0.95 | 0.04 | 0.96 |  |  | 0.05 | 0.95 |
| 30 | 0.05 | 0.95 | 0.05 | 0.95 | 0.04 | 0.96 |  |  | 0.05 | 0.95 |
| 31 | 0.05 | 0.95 | 0.05 | 0.95 | 0.04 | 0.96 |  |  | 0.05 | 0.95 |
| 32 | 0.05 | 0.95 | 0.05 | 0.95 | 0.04 | 0.96 |  |  | 0.05 | 0.95 |
| 33 | 0.05 | 0.95 | 0.05 | 0.95 | 0.04 | 0.96 |  |  | 0.05 | 0.95 |
| 34 | 0.05 | 0.95 | 0.05 | 0.95 | 0.04 | 0.96 |  |  | 0.05 | 0.95 |
| 35 | 0.05 | 0.95 | 0.05 | 0.95 | 0.04 | 0.96 |  |  | 0.05 | 0.95 |
| 36 | 0.05 | 0.95 | 0.05 | 0.96 | 0.04 | 0.96 |  |  | 0.05 | 0.95 |
| 37 | 0.05 | 0.95 | 0.05 | 0.96 | 0.04 | 0.97 |  |  | 0.05 | 0.95 |
| 38 | 0.05 | 0.95 | 0.04 | 0.96 | 0.03 | 0.97 |  |  | 0.05 | 0.95 |
| 39 | 0.05 | 0.95 | 0.04 | 0.96 | 0.03 | 0.97 |  |  | 0.05 | 0.95 |
| 40 | 0.06 | 0.94 | 0.04 | 0.96 | 0.03 | 0.97 |  |  | 0.05 | 0.95 |
| 41 | 0.07 | 0.94 | 0.04 | 0.96 | 0.03 | 0.97 |  |  | 0.05 | 0.95 |
| 42 | 0.07 | 0.93 | 0.04 | 0.96 | 0.03 | 0.97 |  |  | 0.05 | 0.95 |
| 43 | 0.09 | 0.92 | 0.04 | 0.96 | 0.03 | 0.97 |  |  | 0.05 | 0.95 |
| 44 | 0.11 | 0.89 | 0.04 | 0.96 | 0.03 | 0.97 |  |  | 0.05 | 0.96 |
| 45 | 0.16 | 0.85 | 0.04 | 0.96 | 0.03 | 0.97 |  |  | 0.05 | 0.96 |
| 46 | 0.31 | 0.74 | 0.04 | 0.96 | 0.03 | 0.97 |  |  | 0.05 | 0.96 |
| 47 |  |  |  |  | 0.03 | 0.97 |  |  | 0.04 | 0.96 |
| 48 |  |  |  |  | 0.03 | 0.97 |  |  | 0.04 | 0.96 |
| 49 |  |  |  |  | 0.03 | 0.97 |  |  | 0.04 | 0.96 |
| 50 |  |  |  |  | 0.03 | 0.97 |  |  | 0.04 | 0.96 |
| 51 |  |  |  |  | 0.03 | 0.97 |  |  | 0.04 | 0.96 |
| 52 |  |  |  |  | 0.03 | 0.97 |  |  |  |  |
