# Supplementary material for: Comparative Demography of Skates: Life-History Correlates of Productivity and Implications for Management
Source: PLoS One. 2013 May 31;8(5):e65000. doi: 10.1371/journal.pone.0065000 (PMC3669027; doi:10.1371/journal.pone.0065000)
Supplement: Text S1 — Additional caveats and empirical needs. Additional text pertaining to the discussion section Data quality and the status of the best available science in elasmobranch demography. (DOCX) [file pone.0065000.s003.docx]

**Text S1. Additional caveats and empirical needs**

Direct estimates of natural mortality are inherently difficult to obtain for marine populations [1]. All indirect *M* estimation methods used herein were based on age estimates or the parameter estimates obtained from growth models fit to size-at-age data. Thus, if maximum age estimates are incorrect or band pairs are not deposited annually, values of *M* based on those estimates will also be inaccurate. Of the six *M* estimation methods applied, five provide a single estimate for all age classes, with only the Chen and Watanabe [2] method generating age-specific estimates. Although some researchers, e.g. [3], have favored age-specific estimation methods such as the Peterson and Wroblewski [4] approach (using the estimates generated by this method as the most likely values in simulations), we chose to take a more equitable approach by specifying a uniform PDF for mortality-at-age, delimited by the bounds produced by all methods combined. We also chose not to use the Peterson and Wroblewski method because it produces *M* estimates for skates that are substantially lesser than those generated by the other standard methods [5]. Smith et al. [6] found a similar trend in application to the diamond stingray, *Dasyatis dipterura*, and suggested that the method, which was derived from data for arrowworms and small pelagic teleosts, might not be suitable for benthic elasmobranchs. Estimates of *M* applied to the egg stage of each species were based on direct empirical estimates of predation by gastropods [7,8] and represent the most robust estimates for any skate species worldwide. Predation by taxa other than gastropods or mortality related to other causes is not known to contribute significantly to mortality in this stage.

Studies comparing direct and indirect mortality rate estimates have demonstrated that indirect methods often underestimate observed mortality [9,10,11]; therefore, the mortality values incorporated into our analyses may be biased low. On the other hand, most indirect methods of *M* estimation are based on equilibrium conditions and therefore do not account for short-term compensatory responses to changes in fishing mortality (i.e., the theory of density-dependent population growth predicts that *M* values would decrease in response to increased exploitation). Regardless, estimates of *M* produced for all scenarios and age classes varied widely and illustrate the importance of using a Monte Carlo simulation approach to account for these uncertainties.

Analytic theoretical results [12] and the simulations conducted here (Table 6) indicate that incorporating correlation in vital rates among ages decreases the mean and increases the variance of population growth rates, but results in the same inter-specific patterns. Given the large amount of uncertainty in vital rates for many data-poor fish stocks, it may be important to incorporate temporal correlation, depending on the nature of the temporal environmental variation and whether regional abiotic drivers are likely to affect all ages similarly. In most cases, it could be argued that at least some correlation among vital rates is more realistic than assuming that vital rates are iid.

Estimation of annual fecundity is notoriously difficult for oviparious elasmobranch species, particularly those that have protracted or continuous reproductive activity [13], such as skates [14]. Fishery-independent sampling is rarely performed year-round in the EBS due to harsh winter ocean conditions, which precludes the collection of specimens necessary to determine seasonality of parturition, frequency of egg-case formation, or estimation of annual fecundities based on static oocyte counts. Considering these constraints, it was deemed more reasonable to base fecundity estimates on known values for other species. While we believe this is the best possible approach at this time, additional sampling is needed to acquire species-specific fecundity estimates or improve the precision of existing fecundity estimates for particular applications. Regardless, elasticity values indicate that the results reported herein were influenced little by assumptions about fecundity in comparison to survival; therefore, the uncertainty of our estimates is unlikely to invalidate our conclusions.

**References**

1. Vetter EF (1988) Estimation of natural mortality in fish stocks: a review. Fish Bull 86: 25-43.

2. Chen S, Watanabe S (1989) Age dependence of natural mortality coefficient in fish population dynamics. Bull Jpn Soc Sci Fish 55: 205-208.

3. Cortés E (2002) Incorporating uncertainty into demographic modeling: application to shark populations and their convervation. Conserv Biol 16: 1048-1062.

4. Peterson I, Wroblewski JS (1984) Mortality Rate of Fishes in the Pelagic Ecosystem. Can J Fish Aquat Sci 41: 1117-1120.

5. Winton MV (2011) Age, growth, and demography of the roughtail skate, *Bathyraja trachura* (Gilbert, 1892), from the eastern Bering Sea [Master thesis, Moss Landing Marine Labs, California State University Monterey Bay].

6. Smith WD, Cailliet GM, Cortés E (2008) Demography and elasticity of the diamond stingray, *Dasyatis dipterura*: parameter uncertainty and resilience to fishing pressure. Mar Freshw Res 59: 575-586.

7. Hoff GR (2009) Skate *Bathyraja* spp. egg predation in the eastern Bering Sea. J Fish Biol 74: 250-269.

8. Hoff GR (2007) Reproductive biology of the Alaska skate *Bathyraja parmifera*, with regard to nursery sites, embryo development and predation [Dissertation, University of Washington].

9. Cortés E, Parsons GR (1996) Comparative demography of two populations of the bonnethead shark (*Sphyrna tiburo*). Canadian Journal of Fisheries & Aquatic Sciences 53: 709-718.

10. Simpfendorfer CA (1999) Mortality Estimates and Demographic Analysis for the Australian Sharpnose Shark, Rhizoprionodon taylori, from Northern Australia. Fish Bull 97: 978-986.

11. Heupel MR, Simpfendorfer CA (2002) Estimation of mortality of juvenile blacktip sharks, *Carcharhinus limbatus*, within a nursery area using telemetry data. Can J Fish Aquat Sci 59: 624-632.

12. Tuljapurkar S (1990) Population dynamics in variable environments. New York: Springer-Verlag.

13. Barnett LAK, Earley RL, Ebert DA, Cailliet GM (2009) Maturity, fecundity, and reproductive cycle of the spotted ratfish, *Hydrolagus colliei*. Marine Biology 156: 301-316.

14. Holden MJ (1975) The fecundity of *Raja clavata* in British waters. J Cons 36: 110-118.
